# Supplementary figures and images for: Neuronal transcriptome analyses reveal novel neuropeptide modulators of excitation and inhibition imbalance in C. elegans
Source: PLoS One. 2020 Jun 4;15(6):e0233991. doi: 10.1371/journal.pone.0233991 (PMC7272019; doi:10.1371/journal.pone.0233991)

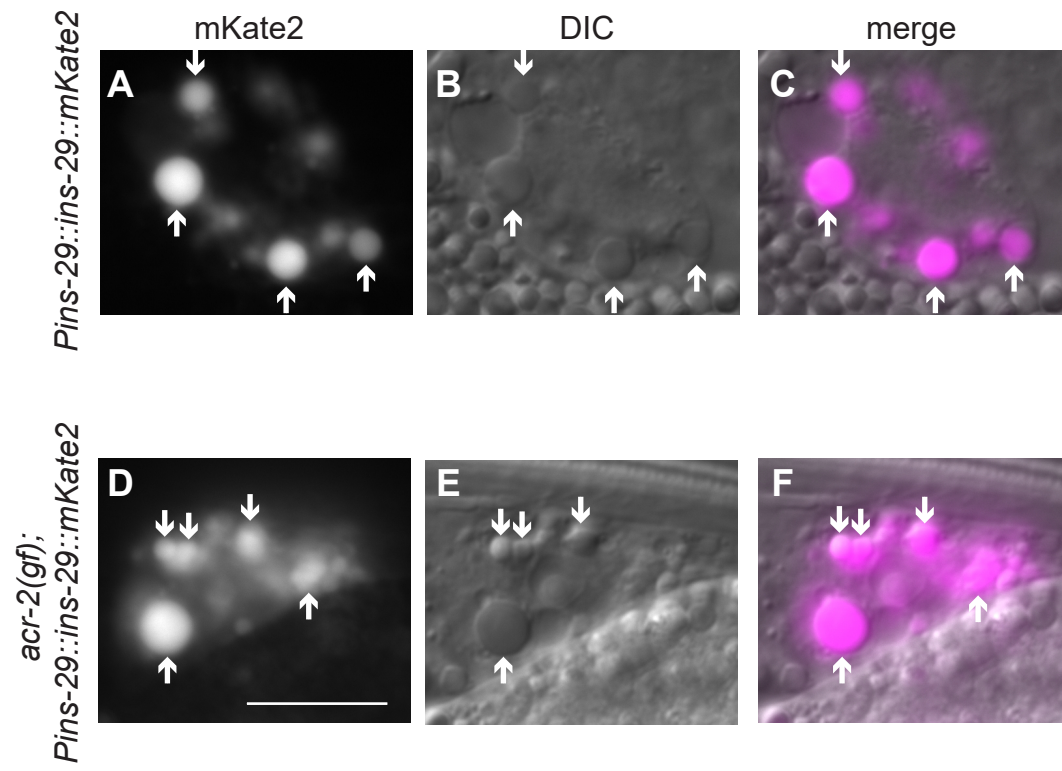

Supplement: S1 Fig — A-F. Shown is expression of directly tagged INS-29 (Pins-29::ins-29::mKate2, juEx8066) in a posterior coelomocyte of D1 adults. (A-C) Shown are fluorescent, differential interference contrast (DIC) and overlay, respectively, of a posterior coelomocyte in wild type carrying the juEx8066 transgene (D-F) Shown are fluorescent, DIC, and overlay images of a posterior coelomocyte in acr-2(gf). Arrows in all images indicate the larger vacuoles in this cell which contain INS-29::mKate2. Scale bar = 10μm. (PDF) [file pone.0233991.s001.pdf]
