# Supplementary material for: Neuronal transcriptome analyses reveal novel neuropeptide modulators of excitation and inhibition imbalance in C. elegans
Source: PLoS One. 2020 Jun 4;15(6):e0233991. doi: 10.1371/journal.pone.0233991 (PMC7272019; doi:10.1371/journal.pone.0233991)
Supplement: S1 Table — (DOCX) [file pone.0233991.s002.docx]

Table S1. Strains and plasmids used in this study

| **STRAIN** | **GENOTYPE** |
| --- | --- |
| MT6241 | *acr-2(n2420)* X |
| XA2260 | *qaIs2240 [Pgcy-33::gfp]* |
| RB2098 | *ins-25(ok2773)* I |
| RB1863 | *flp-12(ok2409)* X |
| RB1340 | *nlp-1* (*ok1469*) X |
| VC1971 | *flp-24(gk3109)* III |
| FX02416 | *ins-6(tm2416)* II |
| OH7160 | *vtIs1 [ Pdat-1::gfp + rol-6(su1006)] V; ets-5(tm866)* X |
| CZ631 | *juIs14*[*Pacr-2::GFP*] IV |
| CZ5808 | *juIs14*[*Pacr-2::GFP*] IV; *acr-2(n2420)* X |
| CZ9299 | *gtl-2(n2618)* IV; *acr-2(n2420)* X |
| CZ25777 | *juEx7741* [*Pins-29::gfp + Pmyo-2::mCherry*] |
| CZ25780 | *acr-2(n2420)* X*; juEx7741* |
| CZ25778 | *juEx7742* [*Pins-29::gfp + Pmyo-2::mCherry*] |
| CZ25781 | *acr-2(n2420) X; juEx7742* |
| CZ25866 | *ins-25(ok2773)* I*; acr-2(n2420)* X |
| CZ25972 | *ins-29 ins-25(ju1580)* I |
| CZ25973 | *ins-29 ins-25(ju1581)* I |
| CZ26034 | *ins-29 ins-25(ju1580)* I*; n2420* X |
| \| CZ26047 \|  \| \| --- \| --- \| | *nlp-1(ok1469) acr-2(n2420)* X |
| CZ26060 | *ins-29 ins-25 (ju1595) ins-27(ok2474) I* |
| CZ26061 | *ins-29 ins-25 (ju1596) ins-27(ok2474) I* |
| CZ26062 | *ins-29 ins-25 (ju1596) ins-27(ok2474) I; acr-2(n2420) X* |
| \| CZ26269 \|  \| \| --- \| --- \| | *flp-12(ok2409) acr-2(n2420)* X |
| CZ26475 | *juEx7879* [*Pnlp-1::gfp + Punc122::mCherry*] |
| CZ26476 | *juEx7880* [*Pnlp-1::gfp + Punc122::mCherry*] |
| CZ26477 | *acr-2(n2420)X; juEx7879* |
| CZ26478 | *acr-2(n2420)X; juEx7880* |
| CZ26817 | *flp-24(gk3109)* III*; acr-2(n2420*) X |
| CZ26818 | *flp-24(gk3109)* III*; nlp-1(ok1469) acr-2(n2420)* X |
| CZ26822 | *flp-24(gk3109)* III; *flp-12(ok2409* X |
| CZ26823 | *ins-29 ins-25(ju1580)* I; *flp-12(ok2409)* X |
| CZ26824 | *flp-24(gk3109*) III; *flp-12(ok2409) acr-2(n2420)* X |
| CZ27016 | *juEx7966* [*Pins-29::ins-29::SL2::mKate2 + Pmec-4::gfp*] |
| CZ26821 | *ins-29 ins-25(ju1580)* I; *flp-12(ok2409) acr-2(n2420)* X |
| CZ27057 | *juEx7965* [*Pflp-12::flp-12::SL2::mKate2 + Pgcy-8::rfp*] |
| CZ27217 | *acr-2(n2420)* X; *juEx7964* |
| CZ27218 | *acr-2(n2420)* X; *juEx7966* |
| CZ27369 | *qaIs2240; juEx7966* |
| CZ27370 | *qaIs2240; acr-2(n2420)* X; *juEx7966* |
| CZ27414 | *juIs14* IV*; juEx7966* |
| CZ27415 | *juIs14* IV; *acr-2(n2420)* X, *juEx7966* |
| CZ27418 | *ins-29(ju1776*) I |
| CZ27419 | *ins-29(ju1776*) I; *acr-2(n2420*) X |
| CZ27420 | *juEx7964 [Pflp-12::flp-12::SL2::mKate2 + Pgcy-8::rfp]* |
| CZ27421 | *juEx7967 [Pins-29::ins-29::SL2::mKate2 + Pmec-4::gfp]* |
| CZ27527 | *ets-5(tm866*) X; *juEx7742* |
| CZ27528 | *ets-5(tm866) acr-2(n2420)* X; *juEx7742* |
| CZ27529 | *gtl-2(n2618)* IV; *juEx7742* |
| CZ27530 | *gtl-2(n2618)* IV; *acr-2(n2420)* X; *juEx7742* |
| CZ27617 | *ins-29 ins-25 (ju1596) ins-27(ok2474*) I; *ins-6(tm2416)* II; *acr-2(n2420)* X |
| CZ27920 | *juEx8064[Pins-29::ins-29::mKate2+Pttx-3::gfp]* |
| CZ27921 | *juEx8065[Pins-29::ins-29::mKate2+Pttx-3::gfp]* |
| CZ27922 | *juEx8066 [Pins-29::ins-29::mKate2+Pttx-3::gfp]* |
| CZ27923 | *acr-2(n2420)* X*; juEx8064* |
| CZ27924 | *acr-2(n2420)* X*; juEx8066* |
| CZ27932 | *ins-29 ins-25 (ju1580)* I*; flp-12(ok2409) acr-2(n2420)* X*; juEx7966* |
| CZ27955 | *juEx8068 [Punc-129::acr-2(gf)::gfp+Pins-29::gfp+Punc-122::mCherry Line#1]* |
| CZ27956 | *juEx8069 [Punc-129::acr-2(gf)::gfp + Pins-29::gfp+Punc-122::mCherry Line#2]* |
| CZ27957 | *juEx8070 [Pgcy-33::acr-2(gf)::gfp + Pins-29::gfp + Punc-122::mCherry Line#1]* |
| CZ27958 | *juEx8071 [Pgcy-33::acr-2(gf)::gfp+Pins-29::gfp + Punc-122::mCherry Line#2]* |
|  |  |
| **Plasmid Name** | **Description** |
| pCZGY1252 | *Punc-129::acr-2(gf)::gfp* |
| pCZ1002 | *Pins-29::gfp* |
| pCZ1003 | *Pnlp-1::gfp* |
| pCZ1004 | *Pflp-12::flp-12::SL2::mKate2* |
| pCZ1005 | *Pins-29::ins-29::SL2::mKate2* |
| pCZ1006 | *Pins-29::ins-29::mKate2* |
| pCZ1007 | *Pgcy-33::acr-2(gf)::gfp* |
