# Supplementary material for: Neuronal transcriptome analyses reveal novel neuropeptide modulators of excitation and inhibition imbalance in C. elegans
Source: PLoS One. 2020 Jun 4;15(6):e0233991. doi: 10.1371/journal.pone.0233991 (PMC7272019; doi:10.1371/journal.pone.0233991)
Supplement: S3 Table — (DOCX) [file pone.0233991.s004.docx]

| Time (Minutes) | | | | | | | |  |
| --- | --- | --- | --- | --- | --- | --- | --- | --- |
| Genotype | 0 | 30 | 60^‡^ | 90 | 120 | 150 | 180 | |
| Wild type | 100 ±0^#^ | 100 ±0 | 100 ±0 | 90 ± 0 | 70±5.7 | 53.3±8.8 | 26.7±3.3 | |
| *acr-2(gf)* | 100±0^†^ | 93.3 ±3.3 | 0±0 | 0±0 | 0 ±0 | 0±0 | 0 ±0 | |
| *flp-2(0) acr-2(gf)* | 100±0 | 86.7±3.3 | 6.7±3.3 | 0±0 | 0±0 | 0±0 | 0±0 | |
| *ins-29(0) ins25(0);*  *acr-2(gf)* | 100±0 | 96.7±3.3 | 6.7±6.7 | 0±0 | 0±0 | 0±0 | 0±0 | |
| *ins-29(0) ins-25 (0);*  *flp-12(0) acr-2(gf)* | 100±0 | 90±5.7 | 10±10 | 3.3±3.3 | 0±0 | 0±0 | 0±0 | |

#Shown are mean ±standard error of the percent animals of each strain at each timepoint that respond to touch on 500µM Aldicarb over three trials. N=10 animals each trial.

†Two-way ANOVA followed by Bonferroni’s post-hoc test was used to compare strains. Compound mutant strains were compared to *acr-2(gf)* at the same timepoint. None were significantly different from *acr-2(gf)* alone.

‡Data is also shown in Figure 6A.
