# Supplementary material for: Neuronal transcriptome analyses reveal novel neuropeptide modulators of excitation and inhibition imbalance in C. elegans
Source: PLoS One. 2020 Jun 4;15(6):e0233991. doi: 10.1371/journal.pone.0233991 (PMC7272019; doi:10.1371/journal.pone.0233991)
Supplement: S4 Table — (DOCX) [file pone.0233991.s005.docx]

| Time (Minutes) | | | | | | | |  |
| --- | --- | --- | --- | --- | --- | --- | --- | --- |
| Genotype | 0 | 30 | 60 | 90 | 120 | 150 | 180 | |
| Wild type | 100 ±0^#,†^ | 100 ±0 | 100 ±0 | 40± 0 | 20±5.7 | 0±0 | 0±0 | |
| *flp-12(0)* | 100±0 | 100 ±0 | 100±0 | 50±0 | 13.3±8.8 | 0±0 | 0 ±0 | |
| *ins-29(0) ins-25(0)* | 100±0 | 100±0 | 100±0 | 46.7±8.8 | 13.3±6.7 | 0±0 | 0±0 | |
| *ins-29(0) ins-25(0);*  *flp-12(0)* | 100±0 | 100±3.3 | 96.7±3.3 | 50±11.5 | 16±6.6 | 0±0 | 0±0 | |

#Shown are mean ±standard error of the percent animals of each strain at each timepoint that respond to touch on 1mM Aldicarb over three trials. N=10 animals each trial.

†Two-way ANOVA followed by Bonferroni’s post-hoc test was used to compare strains. Mutant strains were compared to wild type at the same timepoint. None were significantly different from wild type.
