# Supplementary material for: Neuronal transcriptome analyses reveal novel neuropeptide modulators of excitation and inhibition imbalance in C. elegans
Source: PLoS One. 2020 Jun 4;15(6):e0233991. doi: 10.1371/journal.pone.0233991 (PMC7272019; doi:10.1371/journal.pone.0233991)
Supplement: S5 Table — (DOCX) [file pone.0233991.s006.docx]

| Genotype | 0 | 15^‡^ | 30 | 45 | 60 |
| --- | --- | --- | --- | --- | --- |
| Wild type | 100 ±0^#^ | 96.7 ±3.3 | 76.7 ±3.3 | 33.3 ± 8.8 | 10±0 |
| *acr-2(gf)* | 100±0^†^ | 53.3±6.7 | 16±6.7 | 3.3±3.3 | 0 ±0 |
| *flp-2(0) acr-2(gf)* | 100±0 | 53.3±8.8 | 10±5.7 | 0±0 | 0±0 |
| *ins-29(0) ins-25(0);*  *acr-2(gf)* | 100±0 | 76.7±8.8 | 10±5.7 | 0±0 | 0±0 |
| *ins-29(0) ins-25(0);*  *flp-12(0) acr-2(gf)* | 100±0 | 83.3±8.8* | 10±5.7 | 0±0 | 0±0 |

Time (Minutes)

#Shown are mean ±standard error of the percent animals of each strain at each timepoint that respond to touch on 1 mM Levamisole over three trials. N=10 animals each trial.

†Two-way ANOVA followed by Bonferroni’s post-hoc test was used to compare strains. Compound mutant strains were compared to *acr-2(gf)* at the same timepoint. * p<0.05

‡Data is also shown in Figure 6B.
