# Supplementary material for: Neuronal transcriptome analyses reveal novel neuropeptide modulators of excitation and inhibition imbalance in C. elegans
Source: PLoS One. 2020 Jun 4;15(6):e0233991. doi: 10.1371/journal.pone.0233991 (PMC7272019; doi:10.1371/journal.pone.0233991)
Supplement: S6 Table — (DOCX) [file pone.0233991.s007.docx]

| Genotype | 0 | 15 | 30 | 45 | 60 |
| --- | --- | --- | --- | --- | --- |
| Wild type | 100 ±0^#,†^ | 92.5 ±7.5 | 75 ±12.6 | 45± 12.6 | 15±6.5 |
| *flp-12(0)* | 100±0 | 87.5 ±6.3 | 67.5±9.5 | 22.5±8.5 | 7.5±2.5 |
| *ins-29(0) ins-25(0)* | 100±0 | 92.5±7.5 | 67.5±9.3 | 32.5±12.5 | 15±6.5 |
| *ins-29(0) ins-25(0);*  *flp-12(0)* | 100±0 | 85±9.6 | 62.5±17.5 | 37±13.1 | 15±6.5 |

Time (Minutes)

# Shown are mean ±standard error of the percent animals of each strain at each timepoint that respond to touch on 1mM Levamisole over three trials. N=10 animals each trial.

† Two-way ANOVA followed by Bonferroni’s post-hoc test was used to compare strains. Mutant strains were compared to wild type at the same timepoint. None were significantly different from wild type.
