# Supplementary material for: Neuronal transcriptome analyses reveal novel neuropeptide modulators of excitation and inhibition imbalance in C. elegans
Source: PLoS One. 2020 Jun 4;15(6):e0233991. doi: 10.1371/journal.pone.0233991 (PMC7272019; doi:10.1371/journal.pone.0233991)
Supplement: S7 Table — (DOCX) [file pone.0233991.s008.docx]

Time (Minutes)

| Genotype | 0 | 15^‡^ | 30 | 45 | 60 |
| --- | --- | --- | --- | --- | --- |
| Wild type | 100 ±0^#^ | 66.7±14.5 *** | 36.7 ±12.0 *** | 0± 0 | 0±0 |
| *juEx7966* | 100±0 | 86.7 ±8.8 *** | 78.7±5.9 *** | 7.0±3.5 | 0±0 |
| *acr-2(gf)* | 100±0^†^ | 33.3±8.2 | 0±0 | 0±0 | 0±0 |
| *acr-2(gf); juEx7966* | 100±0 | 30±15.3 | 6.7±3.3 | 0±13.1 | 0±6.5 |
| *ins-29(0) ins-25(0);*  *flp-12(0) acr-2(gf)* | 100±0 | 66.7±12.0 *** | 0±0 | 0±0 | 0±0 |
| *ins-29(0) ins-25(0); flp-12(0) acr-2(gf);*  *juEx7966* | 100±0 | 26.7±8.8 | 0±0 | 0±0 | 0±0 |

# Shown are mean ±standard error of the percent animals of each strain at each timepoint that respond to touch on 1mM Levamisole over three trials. N=10 animals each trial.

† Two-way ANOVA and Bonferroni multiple comparisons were used to compared strains. Shown are comparisons

to *acr-2(gf)* (***P<0.001).

‡ Data is also shown in Figure 6C
