# Supplementary material for: Neuronal transcriptome analyses reveal novel neuropeptide modulators of excitation and inhibition imbalance in C. elegans
Source: PLoS One. 2020 Jun 4;15(6):e0233991. doi: 10.1371/journal.pone.0233991 (PMC7272019; doi:10.1371/journal.pone.0233991)
Supplement: S8 Table — (DOCX) [file pone.0233991.s009.docx]

| GENE | ALLELE | SEQUENCE |
| --- | --- | --- |
| *ins-25* | *ok2773* | Forward 5’cgttgggaaaagtcttgagg3’, Reverse 5’ accaaaacctgaaatggcac3’ |
| *ins-29* | *ju1776* | Forward 5’ cgcccacttttgacctattc 3’, Reverse 5’ gcaagatttgaaggacagcac 3’ |
| *ins-27* | *ok2474* | Forward 5’ggtatctggcaccaggatgag 3’, Internal 5’ tcttccgcttaatcttgctctg 3’, Reverse 5’ ttcagatttgtaggctcacttg 3’ |
| *ins-6* | *tm2416* | Forward 5’ctagcaactccccgtactca3’, Reverse 5’ ggaaagagaacgcactgctc 3’ |
| *nlp-1* | *ok1469* | Forward 5’ gaaacattgtgctccaccct 3’, Reverse 5’ attcagaagcggaaagagca 3’ |
| *flp-24* | *gk3109* | Forward 5’cgatgttccgctctgagcttc3’, Reverse 5’ tggtcacagtgcattgctctc 3’ |
| *flp-12* | *ok2409* | Forward 5’ tgcattttaggaactcgtct 3’, Reverse 5’ tctcgttctcgtttctgatt 3’ |
| *ins-29 ins-25* | *ju1580* | Forward 5’ cgcccacttttgacctattc 3’, Internal 5’ gcaagatttgaaggacagcac 3’, Reverse 5’ ctttgaagttcgcccacagt 3’ |
| *ets-5* | *tm866* | Forward 5’ ggtccatcgctgacctgtat 3’, Internal 5’ taggatgctgccacattgtc 3’, Reverse 5’ aattggttgacccagacgag 3’ |
| *gtl-2* | *n2618* | Forward 5’ gttgttcagattgccaggac 3’, Reverse 5’ acagacaacgagtgaacatc 3’ |
