# Supplementary material for: Neuronal transcriptome analyses reveal novel neuropeptide modulators of excitation and inhibition imbalance in C. elegans
Source: PLoS One. 2020 Jun 4;15(6):e0233991. doi: 10.1371/journal.pone.0233991 (PMC7272019; doi:10.1371/journal.pone.0233991)
Supplement: Attachment — Submitted filename: Response to reviewers.docx [file pone.0233991.s010.docx]

**Authors’ response to review (shown in black)**

**REVIEWER 1:**

*The logic of the findings is not making sense, as the induced hyperactivity of the cholinergic system would be expected to induce upregulation of systems that can counterbalance this hyperactivity, i.e. classical homeostatic responses to allow maintaining somewhat normal functionality of the nervous system. However, knock out of the upregulated neuropeptide genes caused a reduction of the convulsions rather than further increase. The authors should comment on this paradox in the discussion, particularly as overexpression also caused reduction of convulsion (which is more in line with expectations).*

**Response:** We agree with the reviewer that in wild type animals with normal operating nervous system, classical homeostatic responses should allow maintaining somewhat normal functionality of the nervous system. However, *acr-2(gf)* causes abnormal hyperactivity of locomotor circuit, and in the *acr-2(gf)* mutants, normal homeostatic regulation is insufficient to counterbalance overexcitation. We previously reported that convulsions in *acr-2(gf)* animals are reduced when release of dense core vesicles is blocked using genetic null mutations of *unc-31/CAPS [**1**]*. Yet, dense core vesicles can release different types of neuropeptides that exert inhibitory or excitatory effects. Indeed, our investigation of individual peptides has implicated roles of both types of neuropeptides that modulate *acr-2(gf)*-induced convulsion. In the revised manuscript, we have made text revision in the discussion section to address the paradox raised by the reviewer (pg13, paragraph two of the discussion):

“ILPs were the most up-regulated genes identified by RNA-seq in *acr-2(gf)*. We have previously shown that loss of function mutations in *unc-31*/CAPS, which is required for the release of the majority of neuropeptides, reduce convulsion rate of *acr-2(gf*) mutants, indicating that the release of unidentified neuropeptides modulates motor circuit hyperactivity[1,2]. Here, we show that loss of the ILP genes *ins-29* and *ins-25*, along with *flp-12*, can reduce convulsion of *acr-2(gf)* animals. This effect is modulatory, as mutation of these neuropeptides partially suppresses, but does not block, convulsion. The effects of these peptides are in contrast to our previous studies on a different neuropeptide *flp*-18, which is also up-regulated in cholinergic motoneurons of *acr-2(gf)* (this study), yet loss of *flp-18* function, together with loss of *flp-1,* increased convulsion of *acr-2(gf)* animals [1]. These data are consistent with many studies showing that neuropeptide modulation involves combinatorial and redundant action of different peptides that can have varied effects on epileptic states [3]. Paradoxically, we further find that overexpression of wild type *ins-29* or *flp-12* in *acr-2(gf)* reduced convulsion. We speculate that the observed effects from overexpressing each peptide reflect promiscuity of receptors for the neuropeptides. Indeed, the variety of effects observed by *ins-29* over-expression would be consistent with it affecting multiple pathways. Future studies will involve defining specific receptors for each peptide to clarify their function.”

*Altering the function of neuropeptide signaling often results in surprising findings that are not easily reconciled. At stage, the work is premature and asks for more experiments to understand the precise nature of the phenotypes. Possibly, the imbalance in the motor nervous system is more complex due to the effects on GABAergic signaling, which is usually co-stimulated when cholinergic neurons are active. Also, have the authors considered that genes downregulated may have an important role here, e.g. because they are normally promoting (cholinergic) nervous system function?*

**Response:** We appreciate the reviewer’s comment on the complexity of the imbalance in the motor nervous system. Our main goal of this manuscript is to share with the research community our transcriptome analyses in a timely manner. We did not pursue in-depth studies of the genes downregulated in *acr-2(gf)* because most genes are not easily interpretable based on available information; and the full list is presented in supplementary data 2, which we believe is valuable information for researchers with common interests.

*Specific points;*

*The authors say that they analyzed the expressome of adult cholinergic neurons. This is imprecise, because they used the acr-2 promoter-driven GFP to isolate cells for RNAseq. However, while acr-2 is expressed in many cholinergic motor neurons, it is also expressed in numerous unidentified cells, which are not necessarily cholinergic. For example, BAG, in which the INS-29 neuropeptide is overexpressed (as well as ACR-2), is a glutamatergic neuron (Pereira et al., 2015, eLife). Thus, the wording should be adjusted.*

**Response:** Thank you for the suggestion. We used the transgene *juIs14 (Pacr-2::gfp)* for sorting neurons from one-day old adults. We have adjusted the language throughout the manuscript (particularly in the earlier results section, pgs. 6-7), to highlight that we are analyzing cells expressing gfp driven by *Pacr-2*, which includes, but may not be limited to, ventral cord cholinergic neurons.

*The authors show that acr-2 is expressed in BAG neurons, and that acr-2 expression itself is upregulated in BAG in the acr-2(gf) background. This overexpression is surprising – why would the intrinsic acr-2 locus respond to hyperactivity of its own product by upregulation? One would rather expect a downregulation. In this context: Does ACR-2 activity in BAG lead to increased INS-29 release due to depolarization of BAG? Upregulation of a peptide generally counteracting cholinergic hyperactivity, but in a neuron outside the cholinergic system, implies that some signal from these motor neurons is released in response to their hyperactivity, which is sensed in BAG and leads to upregulation of INS-29. This would then lead to increased release of INS-29 peptides. However, if ACR-2 itself is expressed in BAG neurons, increased INS-29 release may not be in response to acr-2(gf) in the cholinergic system but simply because acr-2 is more active in BAG and the cell is more depolarized. To test this the authors could knock down acr-2(gf) in BAG neurons, cell-specifically, and test if these animals still show compensatory activity of ins-29.*

**Response:** We appreciate reviewer’s very thoughtful comment. Indeed, we were surprised by this finding, although we also had no basis to expect a down-regulation of *acr-2*. A recent publication from the Ringstad lab reported the BAG neuron transcriptome[4]. From their dataset, we found that expression of *acr-2,* and *acr-12*, a co-subunit of the ACR-2R was detected, suggesting that BAG neurons may be capable of assembling functional ACR-2R. However, we would like to emphasize our finding (shown in Figure 4H) that the up-regulation of *ins-29* in *acr-2(gf)* was completely eliminated by loss of function in *gtl-2*, which completely suppressed *acr-2(gf)* phenotypes and regulates motor circuit activity imbalance cell non-autonomously through a systematic mechanism [5]. This finding would support a conclusion that the increased expression of *ins-29* is not a result of direct effects of neuronal transmission in BAG.

Additionally, we performed the following experiments. We asked how cell-specific expression of *acr-2(gf)* using cDNA driven by *Pgcy-33* BAG-specific promoter or by *Punc-129* in a small set of cholinergic motor neurons would affect *Pins-29* reporter expression in BAG neurons. We generated transgenic animals expressing P*ins-29* reporters with either *Pgcy-33::acr-2(gf)::gfp* or *Punc-129::acr-2(gf)::gfp. Punc-129* drives expression in a subset of cholinergic motoneurons, and animals expressing *Punc-129::acr-2(gf)::gfp* was previously shown to induce convulsion [6]. As shown in the revised Figure 4I, *acr-2(gf)* expressed under the *Punc-129* promoter promotes convulsion as previously described, but *acr-2(gf)* expressed under the *Pgcy-33* promoter does not [6]. We found that Pins-2::GFP expression is strongly expressed in both BAG neurons in animals expressing *Punc-129::acr-2(gf)* in the motor circuit, but is typically expressed in 1 or no BAG neurons in those animals expressing *Pgcy-33::acr-2(gf)* in the BAG neurons (Figure 4J). Together with the *gtl-2(0)* data, this new data further supports that *acr-2(gf)* hyperactivity in the motor circuit, not BAG neurons, regulates *ins-29* expression. We have made text revision to address reviewer’s points (pg. 10).

*Minor points:*

*In Fig 5C, most animals of ins-29 ins-25(0); flp-12(0); acr-2(gf) were paralyzed. However, they were almost non-paralyzed in Table S3 (60 min time point).*

**Response:** Thank you for catching the typos. Table S3 has been corrected.

*In Fig. 4B, there is expression of gcy-33 in the dorsal cord. This is surprising as gcy-33 expression was previously described only in BAG, URX, AQR and PQR – do the authors know which cells these are?*

**Response:** We apologize for the confusion. This GFP expression is a co-injection marker *Pmec-4::gfp* that labels mechanosensory neurons. For confocal imaging, we mounted the animals such that the top of the field of view is the side of the animal, which also brings Pmec-4::GFP expressing cells in the same view. We have clarified this in the figure legends.

*In Fig 5A, flp-24(0); nlp-12(0) and ins-29 ins-25(0); flp-12(0) partially blocked acr-2(gf). Did they affect cholinergic function also in a normal nervous system (i.e., without acr-2(gf)?*

**Response:** To address the function of these peptides in cholinergic transmission in the absence of *acr-2(gf),* we performed aldicarb and levamisole sensitivity assays. *ins-29 ins-25(0)* and *flp-12(0)* do not affect aldicarb or levamisole sensitivity on their own as shown in supplementary tables 4 and 6. As discussed in the next comment, we show that over-expression of *ins-29* in wild type increases resistance to levamisole. Therefore, at least in wild type context, artificial over-expression of this neuropeptide alone can affect muscle responsiveness.

*In Fig 5D, can the phenotype of ins-29 ins-25(0); flp-12(0); acr-2(gf) be rescued by flp-12 or ins-29 overexpression?*

**Response:** Thank you for the suggestion. We have separated the pharmacology data into a new figure, Figure 6, and updated the results section (pg 11-12) to include this experiment. Over-expression of *ins-29* can revert the levamisole sensitivity of *ins-29 ins-25(0); flp-12(0) acr-2(gf)* animals to that of *acr-2(gf*) alone (Figure 6C). Additionally, over-expression of *ins-29* in wild-type resulted in a levamisole-resistance phenotype. This was interesting given that over-expression of *in-29* alone rescued convulsion, and the effect was similar in the *ins-29 ins-25 (0); flp-12(0) acr-2(gf)* background (Figure 5D). These results together indicate that the over-expression of *ins-29* can be inhibitory or excitatory depending on the context (i.e. genetic background and assay).

**REVIEWER 2**

*However, the authors did not examine how the ins-29 up-regulation is relevant to the phenotype of acr-2(gf).The authors bad better show the following points.*

*1. Where (in which neurons) is the ins-29 originally expressed?
The transgene ins-29p::gfp (or other reporters) can address this question.*

**Response:** Both *ins-29* promoter-driven reporter and full-length *ins-29* with SL2-mKate2 show that *ins-29* is weakly and sporadically expressed in BAG neurons of wild type animals, and is up-regulated in *acr-2(gf)* animals. This was shown in the original Figures 3 and 4.

*2. Is the up-regulation of ins-29 expression responsible for the phenotype of acr-2(gf)?
The gene disruption of ins-29 (using the tm1922 mutant) in acr-2(gf) can easily address this question. In addition, in the double mutant above, a rescue experiment of ins-29 in a BAG neurons-specific manner can enforce the responsibility of ins-29.*

**Response:** Up-regulation of *ins-29* ALONE is not responsible for the phenotype of *acr-2(gf),* because wild type animals with *ins-29*(OE) do not show convulsion. We clarify that up-regulation of *ins-29* is one of the consequences of altered motor circuit by *acr-2(gf).* Single loss of function mutation of *ins-29*, *ins-25*, or *flp-12* does not affect convulsion of *acr-2(gf),* but removing all three neuropeptide genes together cause a reduction of convulsion. Much of the single-mutant data was previously included as a supplemental figure, and we now present the data as Figure 5B in the revised manuscript. These data together (now in Figure 5B,C) support a conclusion that these peptides promote the *acr-2(gf)* phenotype in a redundant fashion.

In the original manuscript, as shown in Figure 5D, we also presented the experiment suggested by the reviewer, which shows that over-expression of *ins-29* in *acr-2(gf)* animals, using its endogenous promoter from high-copy extrachromosomal arrays (driving expression in BAG as shown in Figures 3,4), causes a dominant effect on convulsion rate. Therefore, over-expression of *ins-29* alone in *acr-2(gf)* can affect behavior.

3. *Is the ins-29 translated and secreted after over transcription in BAG neurons under the acr-2(gf) background? The transgene acr-2p::ins-29::mcherry can address this question.
   Otherwise, the authors should explain how the over transcription of ins-29 itself induce the phenotype of theacr-2(gf) mutant*

**Response:** To address this point, we constructed a new transgene, *Pins-29::ins-29::mKate2*, such that the mKate2 reporter is in-frame fused at the C-terminus of INS-29. In the transgenic animals, we observed punctate mKate2 fluorescent signal in the coelomocytes in both wild type and *acr-2(gf)*, indicating secretion of INS-29::mKate2 fusion protein (Figure S1). Text of results of this experiment is in the manuscript (pg. 10). This suggests INS-29 could act on distant cells/tissues in the animal via secretion from head neurons.

*Relevant references:*

**1. Stawicki TM, Takayanagi-Kiya S, Zhou K, Jin Y (2013) Neuropeptides function in a homeostatic manner to modulate excitation-inhibition imbalance in C. elegans. PLoS Genet 9: e1003472.**

**2. Speese S, Petrie M, Schuske K, Ailion M, Ann K, et al. (2007) UNC-31 (CAPS) is required for dense-core vesicle but not synaptic vesicle exocytosis in Caenorhabditis elegans. J Neurosci 27: 6150-6162.**

**3. Kovac S, Walker MC (2013) Neuropeptides in epilepsy. Neuropeptides 47: 467-475.**

**4. Horowitz LB, Brandt JP, Ringstad N (2019) Repression of an activity-dependent autocrine insulin signal is required for sensory neuron development in C. elegans. Development 146.**

**5. Stawicki TM, Zhou K, Yochem J, Chen L, Jin Y (2011) TRPM channels modulate epileptic-like convulsions via systemic ion homeostasis. Curr Biol 21: 883-888.**

**6. Qi YB, Po MD, Mac P, Kawano T, Jorgensen EM, et al. (2013) Hyperactivation of B-type motor neurons results in aberrant synchrony of the Caenorhabditis elegans motor circuit. J Neurosci 33: 5319-5325.**
